# Aqueous ZrO_2_ and YSZ Colloidal Systems through Microwave Assisted Hydrothermal Synthesis

**DOI:** 10.3390/ma6094082

**Published:** 2013-09-16

**Authors:** Kenny Vernieuwe, Petra Lommens, José C. Martins, Freya Van Den Broeck, Isabel Van Driessche, Klaartje De Buysser

**Affiliations:** 1 Department of Inorganic and Chemistry, Ghent University, Krijgslaan 281 S3, B-9000 Ghent, Belgium; E-Mails: kenny.vernieuwe@ugent.be (K.V.); petra.lommens@ugent.be (P.L.); isabel.vandriessche@ugent.be (I.V.D.); 2 Department of Organic Chemistry, Ghent University, Krijgslaan 281 S4, B-9000 Ghent, Belgium; E-Mails: jose.martins@ugent.be (J.M.); freya.vandenbroeck@ugent.be (F.V.D.B.)

**Keywords:** microwave assisted synthesis, YSZ, zirconia, nanoparticles, NMR

## Abstract

In this paper, the formation of ZrO_2_ and yttria-stabilised-zirconia (YSZ) aqueous colloidal systems via microwave assisted hydrothermal synthesis is studied. Microwave synthesis allows a fast screening of the influence of different parameters such as time and temperature. The temperature varied from 140 °C up to 180 °C and the used reaction time varied from 5 min up to 1 h. The synthesised zirconia nanoparticles have a particle size of 50 nm confirmed by TEM. A ^1^H NMR (nuclear magnetic resonance) study helped to understand the stabilization mechanism of the synthesised particles. By the addition of ytrrium ions into the zirconia colloidal solution, YSZ could be formed via an additional thermal treatment. Hereby, the samples are heated up to 400 °C for 1 h. YSZ colloidal solutions are synthesised by making use of complexing agents such as nitrilotriacetic acid, ethylenediaminetetraacetic acid and citric acid to control the hydrolysis and condensation of both ions to avoid non-stoichiometric phases. The ratio of Zr/Y in the particles is quantified by XRF. The amorphous structure of those particles necessitates an additional thermal treatment up to 600 °C during 1 h in order to obtain crystalline YSZ.

## 1. Introduction

Recently, ceramic nanostructured materials gained more and more interest because of the possibility to tune the material properties such as the band gap according to the particle size. Furthermore, the nanosized grains may reduce the sinter temperature. Colloidal precursors make it possible to reduce the processing temperature of thin films because the final sintering step can be avoided or can be performed at reduced temperature [1,2,3,4]. These colloidal solutions are very promising towards green chemistry and energy efficient processing of thin films. Especially, the deposition of crystalline ceramic thin films on polymers or other temperature sensitive substrates via chemical solution deposition will open a novel branch of applications in the near future. The synthesis of crystalline TiO_2_ thin films via precursors containing TiO_2_ particles at temperatures below 300 °C has already been achieved and proves the possibility of low temperature synthesis of ceramics [5,6,7].

In this paper, the synthesis of colloidal YSZ solutions is explored. Pure zirconia undergoes several phase transformations when heated or cooled [8]. At room temperature, a monoclinic crystal structure is obtained. A phase transformation from monoclinic to tetragonal crystal structure occurs at 1170 °C and at 2370 °C the tetragonal phase transforms into a cubic crystal structure. Stabilisation of the cubic structure at room temperature guarantees good mechanical, thermal and electrical properties [9,10]. This is possible by doping ZrO_2_ with MgO, Y_2_O_3_ or CaO [11,12]. When 8 mol% Y_2_O_3_ is integrated in ZrO_2_ (8YSZ), the cubic crystal structure can be maintained at room temperature till melting point [9]. Due to the very low thermal conductivity (2.2 W/(m·K) at 800 °C) 8YSZ can also be used as a thermal barrier coating [13]. Next to the low thermal conductivity, 8YSZ is an excellent oxygen ion conductor at high temperatures. Hereby 8YSZ can act as an electrolyte layer in solid oxide fuel cells. Unfortunately, high temperatures (1200 °C or more) are required to obtain dense YSZ thin films [14,15,16]. High temperatures and long sinter processes are energy consuming and can introduce interactions with the support membrane, which is unfavourable for high quality electrolytes [9,17]. The synthesis of stable ZrO_2_ and YSZ nanoparticles in aqueous solutions that can be deposited as a thin film in a further stage by dip-coating [16], spin-coating [18], or even ink-jet printing [19] is the focus of this paper. This approach can lead to a reduction in sinter temperature and improvement in the homogeneity of the synthesised electrolytes. Synthesizing a ZrO_2_ colloidal solution is the first step towards the synthesis of YSZ nanoparticles. According to Okubo *et al.* [4], there are two ways to obtain YSZ ceramics starting from colloidal solutions: (1) When the interparticle approach is applied, the surface of the zirconia nanoparticles is modified with yttrium ions. Due to the short diffusion path of yttrium (in this case the zirconia particles are up to 15 nm) it is expected to synthesise the mixed oxide at low temperatures; (2) an alternative approach is the intraparticle route. During synthesis of these nanoparticles, yttrium as well as zirconium will undergo hydrolysis and condensation reactions. Finally, YSZ particles will be formed. As zirconium ions are very sensitive for hydrolysis, the formation of YSZ nanoparticles can be impeded by non-stoichiometric reactions, resulting in secondary phases or single metal oxides [4,20]. These secondary phases can be avoided by the addition of coordinating ligands. The ligands interact with the metal ions and form zirconium and yttrium complexes that prevent the synthesis of non-stoichiometric compounds.

The nanoparticles will be synthesised in a more environmental friendly manner. Water is favoured as solvent and lower reaction temperatures and shorter reactions times are addressed to reduce the energy consumption [21]. Compared to conventional methods such as hydrothermal treatments and hot-injections reactions, microwave assisted synthesis has several benefits. The more efficient heating mechanism of microwave irradiation allows lower synthesis temperatures and/or shorter reaction times accompanied by higher yields [22,23,24]. Also, homogenous heating is obtained without suffering from thermal gradient effects. This is beneficial towards industrial production of high quality nanomaterials. The modern microwave equipment allows a fast screening of several parameters due to the shorter reaction times [22,25].

## 2. Results and Discussion

### 2.1. Synthesis of ZrO_2_ Particles

The synthesis of a ZrO_2_ colloidal solution is the first step towards the formation of YSZ ceramics via the interparticle route. As described in the experimental section, zirconium oxynitrate is selected as precursor. When it’s dissolved in water, a cyclic tetramer is formed, [Zr_4_(OH)_8_(OH_2_)_16_]^8+^, in which Zr atoms are eightfold coordinated by four OH– groups and four water molecules [26]. The tetramer complexes play an important role in the formation of ZrO_2_ nanoparticles, by heating the aqueous solution, the tetramer complexes will deprotonate as shown in Equation (1) [27]:

[Zr_4_(OH)_8_(OH_2_)_16_]^8+^ ↔ [Zr_4_(OH)_(8 + *x*)_(OH_2_)_(16 − *x*)_]^(8 − *x*)+^ + *x* H^+^ (1)

As illustrated by Matsui *et al*. [27], the deprotonated tetramer starts to polymerise and when supersaturation of these polymeric species occurs, nuclei are formed. Finally, agglomeration occurs and secondary particles are formed consisting out of several primary particles.

Microwave hydrothermal treatment of solution A at different reaction temperatures and times resulted in ZrO_2_ suspensions with a variety of particle sizes (Table 1.). Synthesis at temperatures above 160 °C resulted in precipitation. These precipitates could also be found in synthesis procedures that took longer than 5 min at 160 °C. Several washing steps are introduced to purify the synthesised colloidal systems for further characterisation.

**Table 1:** Overview of microwave treated solution A where the temperature and duration of the synthesis are represented in function of the particle size and crystallinity.

Temperature (°C)	Duration (min)	Particles size ^[a]^ (nm)	Crystalline	Present phase	Crystallite size (nm)
140	5	37	No	–	–
140	60	47	Yes	Monoclinic ZrO_2_	3.13 ± 0.11
150	5	45	Yes	Monoclinic ZrO_2_	2.64 ± 0.11
150	30	52	Yes	Monoclinic ZrO_2_	3.37 ± 0.10
160	5	50	Yes	Monoclinic ZrO_2_	3.35 ± 0.10

^[a]^ Secondary particle size is measured by DLS (dynamic light scattering).

**Figure 1:**
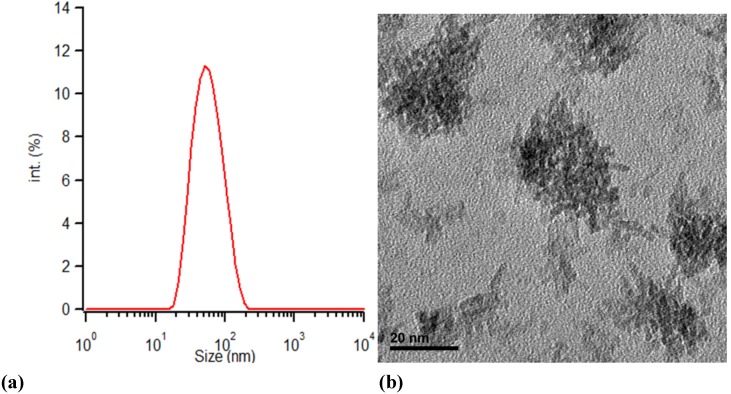
(**a**) The particle size of ZrO_2_ particles synthesized by microwave treatment of solution A at 150 °C for 30 min measured by DLS; and (**b**) TEM micrograph of the solution A treated for 30 min at 150 °C.

As illustrated in Figure 1, TEM and DLS measurements of a sample synthesized at 150 °C for 30 min agree about the particles size considering that TEM measurements show the real particle size while DLS measures the hydrodynamic radius of the particles, the synthesized particles meet the expectations of Matsui *et al.* [27]. The primary particles aggregate and form secondary particles of 50 nm diameter. The primary particles are rod shaped while the secondary structures have irregular shapes.

**Figure 2:**
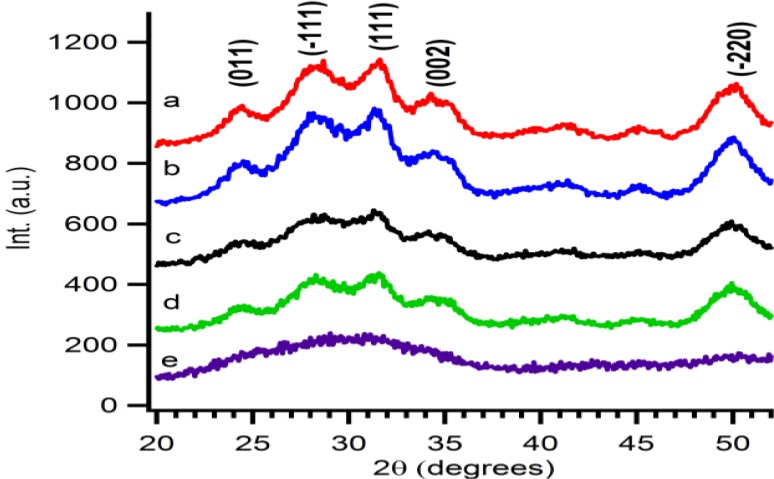
XRD patterns of precipitated particles derived from microwave treated solution A at (**a**) 160 °C for 5 min; (**b**) 150 °C for 30 min; (**c**) 150° C for 5 min; (**d**) 140 °C for 60 min; and (**e**) 140 °C for 5 min.

X-ray diffraction patterns are taken from purified particles which are precipitated and dried for 12 hours at 60 °C. The observed reflections show the presence of Baddeleyite (monoclinic ZrO_2_), pdf card # 36-0420. As shown in Figure 2, the crystallinity of the ZrO_2_ particles is influenced by two parameters, *i.e.*, the temperature and reaction time. The crystallinity of the particles increases with longer synthesis times. At fixed reaction time, for example at 5 min, a temperature influence is visible. Higher temperatures favour the formation of crystalline material. The crystallite size is calculated by a simple Rietveld refinement with the help of *TOPAS-Academic*,* V4.1* software [28]. It is noticed that the crystallite size shows the same trend the crystallinity. Longer reaction times or higher reaction temperatures result in larger crystallite sizes. This can be attributed to higher growth rates of the crystallites at higher temperatures or more time given to the system to grow.

**Figure 3:**
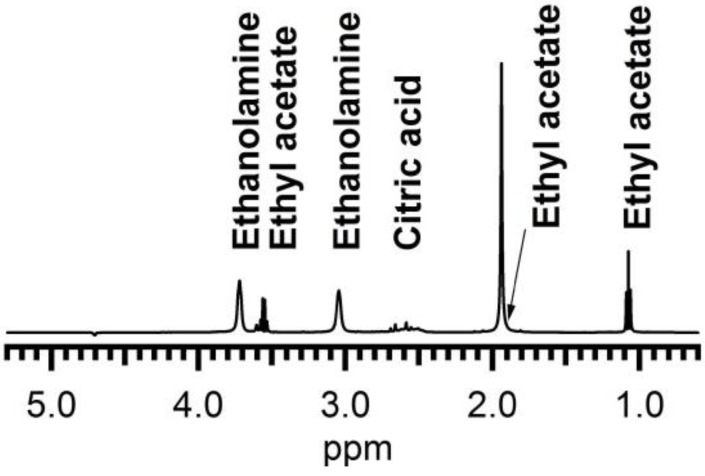
1D proton NMR spectrum of solution A after microwave treatment, purification and the addition of CA and EA.

**Figure 4:**
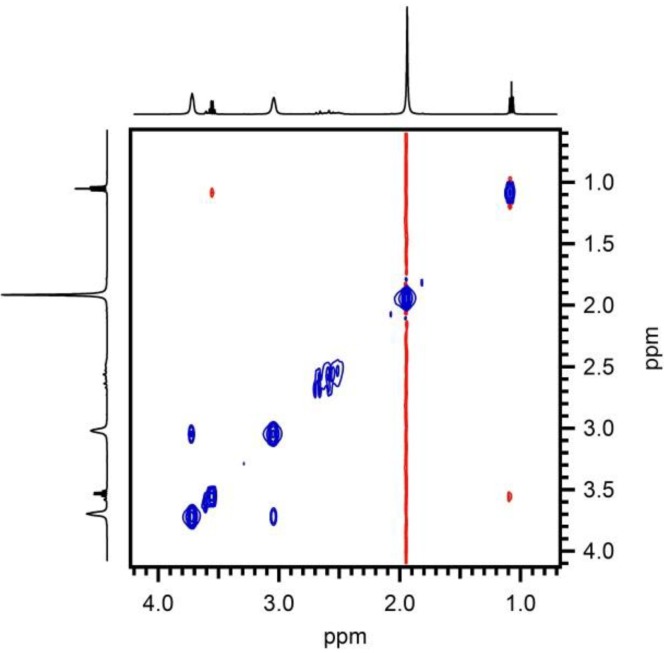
2D NOESY spectrum of solution A microwave treated at 150 °C during 30 min after purification and the addition of CA and EA. Positive and negative NOE cross-peaks appear in red and blue and are attributed to their respective species.

The stabilization of these particles is also investigated. After synthesis the zeta potential has a value of 25 mV that indicates electrostatic stabilization. 1D proton NMR confirmed this hypothesis. After purification PEG1000 was found neither in the solution nor on the particles. Despite the absence of ligands in the colloidal solution, the ZrO_2_ suspension remains stable [29]. As the pH level of this suspension is around 0.9, thus very acidic and not suited for industrial applications, an attempt was made to raise the pH. Higher pH levels make a less corrosive solution and more suited for the ink jet printing system. Precipitation is observed when the pH level is raised by the addition of a base. The formation of the precipitation could be avoided by addition of complexing ligands such as citric acid (CA) before the pH level is raised up to 7. In this case, a stable solution is obtained. Ethanolamine (EA) is used to adjust the pH level because it is a high boiling alkaline component that is harder to evaporate than ammonium hydroxide, which is often used in chemical solution methods. ^1^H NMR spectroscopy was used to investigate the presence of organic ligands in the dispersion and their possible interaction with the ZrO_2_ particles, using recently established methods for semi-conductor colloidal quantum dots [30]. The presence of CA and EA even after washing steps is easily confirmed by noting the presence of their fingerprint in the 1D ^1^H NMR spectrum (Figure 3). The signals at 3.7 and 3.05 ppm can be assigned to each of the methylene groups in EA and appear markedly broadened, a sign of interaction with the surface of a larger particle. The resonances around 2.6 ppm, with sharper lines and scalar coupling fine structure characteristic for small non interacting molecules, are assigned to CA. These appear on top of a broader background that could be attributed to bound CA, in slow or no chemical exchange with the particle surface [31]. Other, equally sharp signals are attributed to ethyl acetate (Figure 3). The 2D NOESY (Nuclear Overhauser Enhancement Spectroscopy) spectrum (Figure 4) displays both strong negative and weak positive NOE (Nuclear Overhauser Enhancement) cross peaks as a result of cross-relaxation by dipolar interaction between proton spins that occur close (<5 A) to one-another in space. As has been discussed in more detail elsewhere [30,31], the fast reorientational dynamics of small molecules in solution causes the slow development of weak and positive NOE cross-peaks. On the other hand, the much slower reorientational dynamics of large molecules in solution results in rapid development of strong and negative NOE cross-peaks. Since the reorientation dynamics of ligands that are bound to the nanoparticle surface will be dominated by the large nanoparticle, strong and negative NOE cross-peaks are expected for any ligand that spends time interacting with the particle surface [30]. Translating this to the case at hand, the following can be concluded. The small positive NOEs visible between the methyl and methylene resonance in the ethylgroup of ethyl acetate indicate that this molecule is free in solution at all times. Between the two EA methylene ^1^H resonances however, large negative NOEs are visible, indicating that EA does interact with the nanoparticle surface and acts as ligand. The occurrence of two sets of signals for CA, one low intensity and broadened and one sharper resonances, suggests CA occurs in two separately visible states. As the resonances of each species are insufficiently spaced, the nature of the NOE contacts cannot be established here. This notwithstanding it appears some CA is also interacting with the surface. Thus EA and most probably also CA contribute to the stabilisation of the dispersion thereby preventing the precipitation from occurring at neutral pH levels.

### 2.2. Interparticle Route towards YSZ

**Figure 5:**
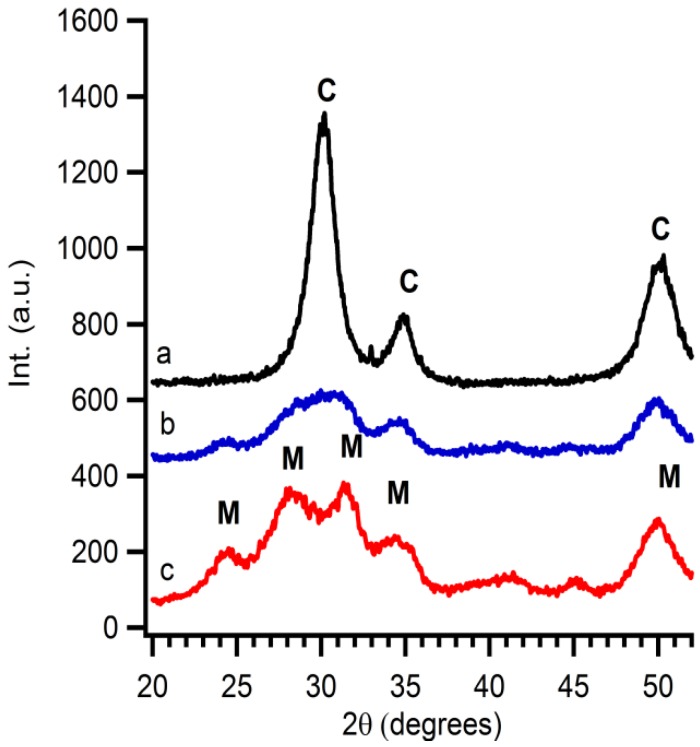
The influence of the temperature on the diffusion of Y into ZrO_2_ nanoparticles shown by XRD measurements (**a**) treated at 400 °C for 1 h; (**b**) treated at 300 °C for 1 h; and (**c**) as-synthesised, M marks the diffraction peaks of monoclinic ZrO_2_; and C the diffraction peaks of YSZ.

**Figure 6:**
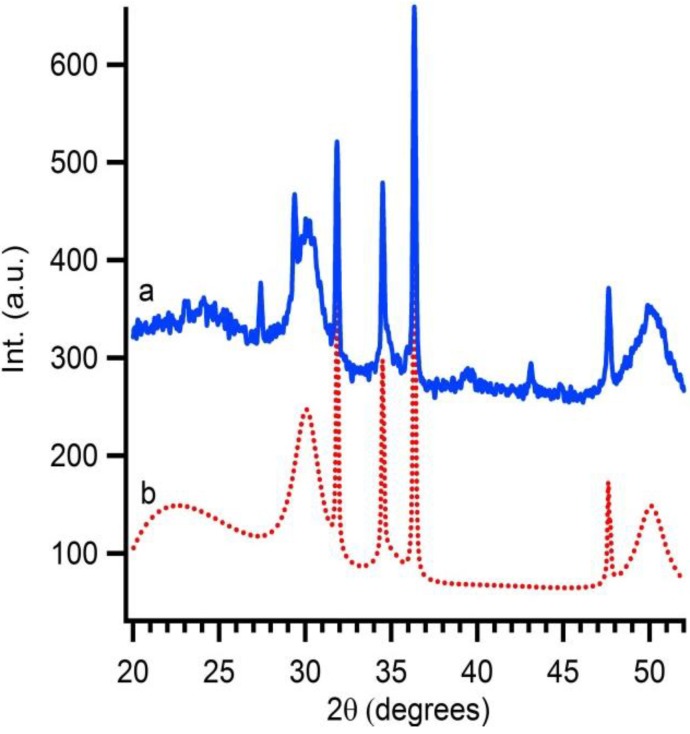
Rietveld refinement of the sample treated at 400 °C for 1 h with (**a**) the experimental data, sample inclusive internal standard; and (**b**) the calculated diffractogram.

When the interparticle route is followed Y(NO_3_)_3_·*x*H_2_O should be added at the ZrO_2_ suspension in the correct stoichiometric conditions. The exact yttrium concentration after addition is checked by XRF. In this case, 16 mol% of yttrium nitrate is added to synthesize 8YSZ at the end. A thermal treatment of this solution gives rise to diffusion of Y into the ZrO_2_ particles resulting in 8YSZ. The conversion from monoclinic ZrO_2_ to cubic YSZ is monitored by XRD taken after each annealing step (Figure 5). Monoclinic zirconia is already observed before calcination. After calcination of the suspension at 300 °C for 1 hour, a slight change in crystal structure is noticed, indicating that a mixture of monoclinic ZrO_2_ and cubic YSZ is obtained. This demonstrates the start of a phase transformation from the monoclinic to cubic crystal structure. Samples calcined at 400 °C during 1 hour only show the presence of 8YSZ. Gibson *et al.* [32] addressed the fact that the difference between nanosized cubic YSZ and tetragonal YSZ is hard to distinguish. Therefore, additional XRD measurements are performed. Because the crystallinity is too low, no reflections are observed in the 2θ region of 72 to 75. Therefore samples are mixed with a 10 wt% internal standard (ZnO). Software is used to perform Rietveld refinement (Topas Academic). The fit includes ZnO, tetragonal and cubic YSZ. The calculated plot with a goodness of fit of 1.18 matches cubic 8YSZ mixed with internal standard fits the experimental data (Figure 6). No secondary phases such as tetragonal YSZ, Y_2_O_3_ or monoclinic ZrO_2_ are detected in the diffraction pattern. This gives proof that the diffusion of dopants into 50 nm colloidal systems is possible and the interparticle route is a good alternative to obtain mixed or doped ceramic oxides at reduced temperatures.

### 2.3. Intraparticle Route towards YSZ

As mentioned before, zirconium ions are very sensitive to hydrolysis compared to yttrium ions. The hydrolysis of zirconium even occurs in strongly acidic solutions and the formation of polynuclear species takes the upper hand. Due to the extremely low solubility of these species (<10^−8^ M), even at low pH ranges, precipitation will appear [20]. Introducing chelating ligands can be a possible solution to lower the hydrolysis rate of the zirconium ions. The ligands can form complexes with both zirconium and yttrium ions. This will lower the amount of free ions in the solution. Hereby, hydrolysis of yttrium and zirconium can be controlled and tuned. Several ligands such as CA, nitrilotriacetic acid (NTA) and ethylene diaminetetraacetic acid (EDTA) were tested. Only the citric acid precursor (solution B) exposed the presence of the correct stoichiometric Y/Zr ratio after microwave treatment and several washing steps. The stoichiometry was determined by XRF measurements. The NTA precursors (solution C) were microwave treated for five to 10 min at 110 °C. Large precipitates were observed but no YSZ was formed. When EDTA precursors (solution D) were microwave treated for 60 min at 180 °C a stable colloidal solution is obtained, however, the quantity of reacted yttrium could not be controlled. XRF measurements showed a large variety in amount of yttrium present in the YSZ particles. The amount of yttrium inside the synthesised particles fluctuates from 0 to 6 mol%, which makes it hard to synthesise 8YSZ nanoparticles in a controlled manner.

**Figure 7:**
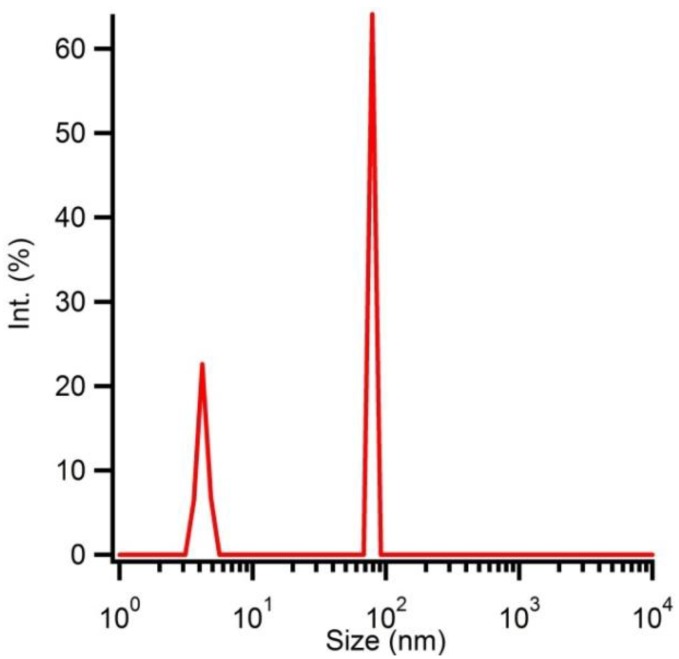
Hydrodynamic radius of solution B microwave treated at 180 °C for 10 min determined by DLS measurements.

**Figure 8:**
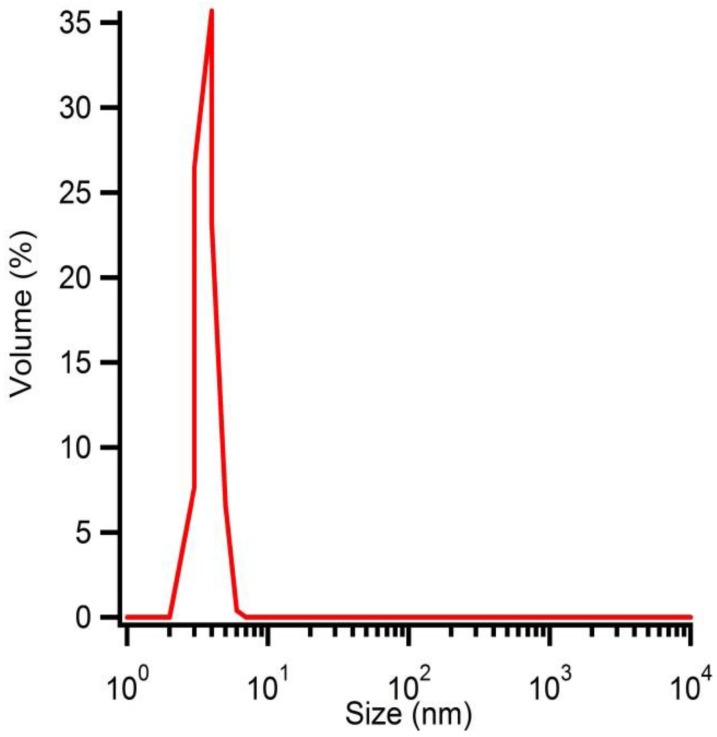
Hydrodynamic radius of solution B microwave treated at 180 °C for 10 min determined by DLS measurement**s **represented as a function of volume distribution.

Solution B at pH 7 is microwave treated during 10 min at 180 °C. After purification and washing, the hydrodynamic radius of the particles is determined by DLS (Figure 7) and polydispersive behaviour is observed. One distribution shows particles with an average hydrodynamic radius of 5 nm while the other distribution shows larger particles, which can be explained by aggregation of the smaller particles. However, a volumetric distribution shows monodisperse behaviour due to the correlation between the observed intensities and the particle volumes (Figure 8). The size distribution is reported by relative intensities. To convert the monitored intensity distribution to a volumetric distribution, the volume of a sphere should be taken into account. This makes the intensity proportional to the cube of the particle diameter. Hereby, we can conclude that the amount of agglomerates is limited and most of the particles have an average hydrodynamic radius of 5 nm.

**Figure 9:**
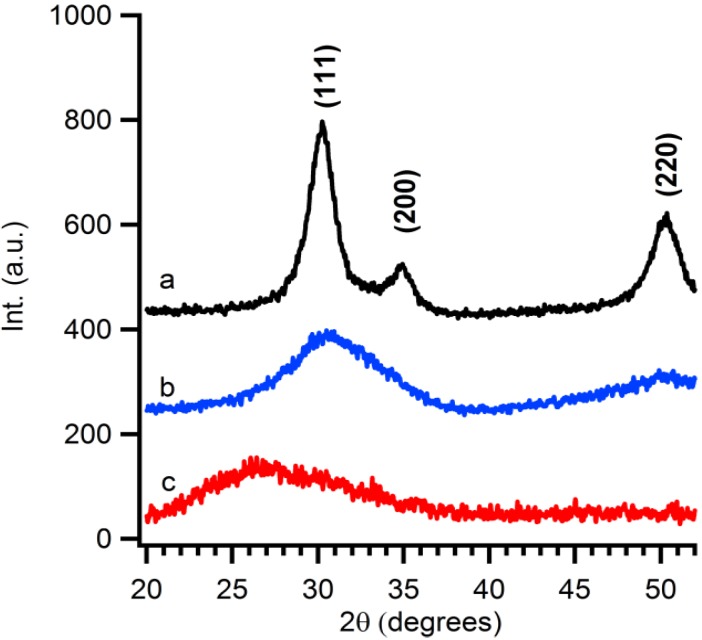
The influence of thermal processing on the crystallinity of the YSZ nanoparticles shown by XRD measurements (**a**) treated at 600 °C for 1 h; (**b**) treated at 400 °C for 1 h; and (**c**) as-synthesised.

**Figure 10:**
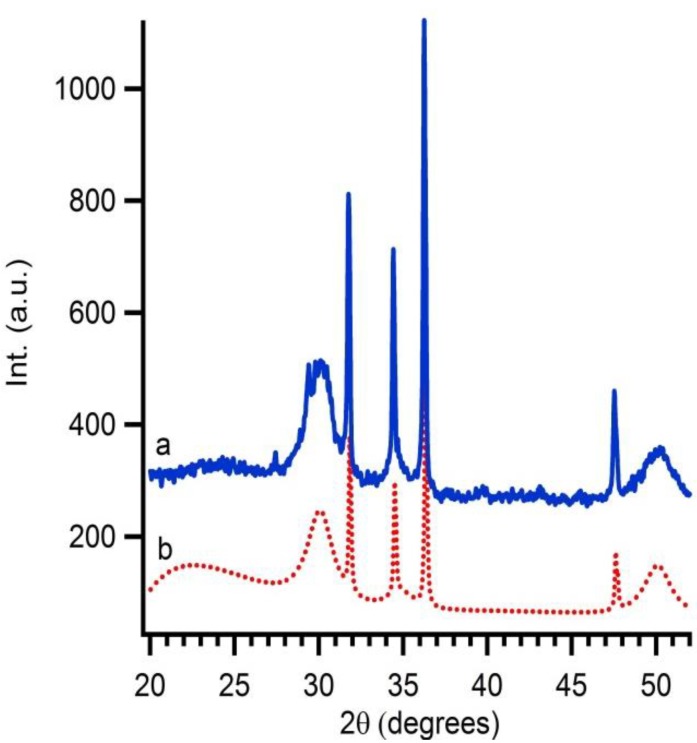
Rietveld refinement of the sample treated at 600 °C with (**a**) the experimental data, sample inclusive internal standard; and (**b**) the calculated diffractogram.

The as-synthesised YSZ particles don’t show any form of crystallinity. An additional thermal treatment transforms this colloidal solution into crystalline powders or thin films. As shown in Figure 9, cubic YSZ is formed at 600 °C. All peaks can be assigned to the cubic phase of 8YSZ, pdf card # 30–1468. Also, in this case Rietveld refinement is performed on the sample which is thermal treated at 600 °C (Figure 10). The fit of the calculated data matches the experimental (goodness of fit 1.21) and the formation of cubic 8YSZ is proven.

The crystallisation temperature of the amorphous particles via the intraparticle route is 200 °C higher than the interparticle approach which makes this a more energy consuming process. When stable crystalline YSZ nanoparticles in aqueous solution could be obtained, the intraparticle approach would be preferred to synthesise thin films. The interparticle route is accompanied by an additional phase transformation as pure zirconia particles are formed in the first step of the process. These monoclinic particles will change to a cubic crystal structure after the interdiffusion of yttrium ions. This phase transformation is associated with a change in crystal lattice dimensions. During thin film processing, a phase transformation is often associated with stresses and cracks [33,34]. Though, the intraparticle route can avoid the diffusion step of the yttrium ions into the zirconia matrix, the obtained amorphous YSZ particles need a higher post-thermal treatment. The intraparticle approach can be most promising if the crystallinity of the YSZ particles could be improved.

## 3. Experimental Section

The microwave assisted synthesis of the aqueous ZrO_2_ precursor starts by dissolving ZrO(NO_3_)_2_•xH_2_O (Sigma Aldrich, St. Louis, MO, USA) in deionised water (0.05 µS/cm at 25 °C). When a clear solution is obtained, 2.5 wt% of PEG 1000 (Alfa Aesar, Ward Hill, MA, USA) is added and a final concentration of 0.33 mol/L Zr^4+^ ions at pH 0.9 is obtained (solution A). Two millilitre of the precursor is treated by a microwave assisted hydrothermal treatment (10 mL glass microwave tube—CEM discover microwave device operating at 2.45 GHz) at different temperatures and reaction times. The suspensions are purified by addition of a non-solvent mixture of ethyl acetate (Carl Roth, Karlsruhe, Germany) and ethanol (Carl Roth). The addition of citric acid (Carl Roth) and ethanolamine (Sigma Aldrich) made it possible to restabilise the ZrO_2_ nanoparticles in water at a neutral pH level after the purification procedure.

For the formation of 8YSZ colloidal systems an adapted precursor solution is needed. By dissolving ZrO(NO_3_)_2_·*x*H_2_O (Sigma Aldrich), Y(NO_3_)_3_·*x*H_2_O (Sigma Aldrich) and citric acid (Carl Roth) in deionised water (0.05 µS/cm at 25 °C) this mixed precursor is obtained (ratio metal ions: citric acid is 1:1). The pH is controlled by addition of ethanolamine (Sigma Aldrich) and/or formic acid (Carl Roth). Finally, the precursor (solution B) contains 0.33 mol/L of Zr^4+^ ions. The microwave treatment converts this precursor into a colloidal system. solution C and D are prepared via similar recipe.

The particle sizes and particle size distributions are examined by dynamic light scattering (Zetasizer Nano ZS, Malvern Instruments, Worcestershire, UK) & TEM (Jeol JEM C_s_-corrected 2200FS, Peabody, MA, USA, operating at 200 kV). Specimens for TEM studies were prepared by adding a drop of purified aqueous sol onto a 300 mesh holey carbon grid. The X-ray diffraction set-up (Thermo Scientific ARL X' tradiffractometer, Waltham, MA, USA, Cu Kα = 1.5405 Å) is used to determine the crystallinity and crystal phases. The samples are measured in a θ–2θ geometry using a 0.02° stepsize and 1 s step couting time. When necessary the internal standard approach was selected to determine the exact crystalline phase. The samples are mixed with 10 wt% ZnO (internal standard) and side loaded to a special sample holder to reduce preferential orientation effects. *TOPAS-Academic V4.1* software was used to perform Rietveld refinement [28]. X-ray fluorescence (Rigaku NEX CG, Tokyo, Japan) is applied to analyze the amounts of yttrium and zirconium in the YSZ samples. NMR experiments such as 1D proton and 2D NOESY (Bruker 500 MHz AVANCE III, Billerica, MA, USA) were performed to identify the presence of coordinating ligands fixed to the particle surface. Towards the NMR measurements, the aqueous sols are mixed with 5 v% D_2_O and measurements are performed using pulse sequences from the standard Bruker library. For both the 1D proton and 2D NOESY, the water signal was suppressed using excitation sculpting. The 2D NOESY was measured using a 300 ms mixing time.

## 4. Conclusions

Via the interparticle route YSZ nanoparticles are obtained. The microwave treated citric acid precursors resulted in amorphous YSZ nanoparticles of 5 nm. Larger aggregates were also formed; with an average size of 200 nm. These amorphous particles are transformed to crystallinie YSZ particles through a thermal treatment at 600 °C during 1 hour. Further research is needed to obtain crystalline YSZ particles without an additional calcination step. In this study, lower fabrications temperatures can be addressed via the interparticle route. It allows the synthesis of YSZ powders at 400 °C. Therefore, aqueous colloidal solutions of monoclinic zirconia nanoparticles are synthesised by microwave treatments at 140–160 °C during 5–60 min. The polymerisation of the tetrameric zirconium species result in the formation of secondary particles with an average size of 30–50 nm. The addition of ethanolamine and citric acid after synthesis helps to stabilise the colloids formed at neutral pH levels instead of 0.9. A ^1^H NMR study resulted in the confirmation that both ligands interact with the particle surfaces and contribute to the stabilisation of the particles. Both ligands contribute to the steric stabilisation of the colloids at neutral pH levels. Finally, crystalline 8YSZ is obtained when 16 mol% of yttrium nitrate hydrate is added to the colloidal solution. A mild thermal process converts the monoclinic zirconia into cubic YSZ.
